# Interleukin (IL)-23 Stimulates IFN-γ Secretion by CD56^bright^ Natural Killer Cells and Enhances IL-18-Driven Dendritic Cells Activation

**DOI:** 10.3389/fimmu.2017.01959

**Published:** 2018-01-17

**Authors:** Andrea Ziblat, Sol Y. Nuñez, Ximena Lucía Raffo Iraolagoitia, Raúl German Spallanzani, Nicolás I. Torres, Jessica M. Sierra, Florencia Secchiari, Carolina I. Domaica, Mercedes B. Fuertes, Norberto W. Zwirner

**Affiliations:** ^1^Instituto de Biología y Medicina Experimental (IBYME-CONICET), Laboratorio de Fisiopatología de la Inmunidad Innata, Buenos Aires, Argentina; ^2^Departamento de Química Biológica, Facultad de Ciencias Exactas y Naturales, Universidad de Buenos Aires, Buenos Aires, Argentina

**Keywords:** natural killer cells, interleukin-23, interleukin-18, IFN-γ, dendritic cells

## Abstract

Interleukin (IL)-23 is a member of the IL-12 family of cytokines that, as the other members of this family, is secreted by monocytes, macrophages, and dendritic cells (DC) upon recognition of bacterial, viral, and fungal components. IL-23 is critical during immunity against acute infections, and it is also involved in the development of autoimmune diseases. Although immunoregulatory effects of IL-23 on mouse natural killer (NK) cells have been described, the effect of IL-23 on human NK cells remains ill-defined. In this study, we observed that monocytes stimulated with LPS secreted IL-23 and that blockade of this cytokine during monocyte and NK cell coculture led to a diminished production of IFN-γ by NK cells. Accordingly, rIL-23-induced NK cell activation and stimulated IFN-γ production by CD56^bright^ NK cells. This effect involved MEK1/MEK2, JNK, PI3K, mammalian target of rapamycin, and NF-κB, but not STAT-1, STAT-3, nor p38 MAPK pathways. Moreover, while NK cell-mediated cytotoxicity remained unaltered, antibody-dependent cellular cytotoxicity (ADCC) was enhanced after IL-23 stimulation. In addition, IL-23 displayed a synergistic effect with IL-18 for IFN-γ production by both CD56^bright^ and CD56^dim^ NK cells, and this effect was due to a priming effect of IL-23 for IL-18 responsiveness. Furthermore, NK cells pre-stimulated with IL-18 promoted an increase in CD86 expression and IL-12 secretion by DC treated with LPS, and IL-23 potentiated these effects. Moreover, IL-23-driven enhancement of NK cell “helper” function was dependent on NK cell-derived IFN-γ. Therefore, our results suggest that IL-23 may trigger NK cell-mediated “helper” effects on adaptive immunity, shaping T cell responses during different pathological situations through the regulation of DC maturation.

## Introduction

Natural killer (NK) cells constitute a subgroup of type 1 innate lymphoid cells that are key players during immunity against intracellular pathogens and tumors due to their cytotoxicity and the secretion of IFN-γ and other pro-inflammatory cytokines (1–3). In humans, they are subdivided into two subpopulations based on the relative expression of CD56 and CD16 (4). Almost 90% of peripheral blood NK cells are CD56^dim^CD16^+^ (CD56^dim^), and although they can produce cytokines upon activation (5, 6), their principal effector function is the cytotoxic activity displayed against susceptible target cells (7, 8). The rest of NK cells in blood are CD56^bright^CD16^dim/−^ (CD56^bright^), which are mainly producers of cytokines (4, 8) and are more abundant in secondary lymphoid organs where they exert immunoregulatory functions (9). NK cells become activated after direct recognition of infected or tumor cells through a vast array of activating receptors (10) and by cytokines such as interleukin (IL)-12, IL-15, and IL-18 (11, 12) or their combination with PAMPs (13). Monocytes, macrophages, and dendritic cells (DC) are the major producers of these cytokines during their crosstalk with NK cells (14–16). As a result of this bidirectional crosstalk, in some circumstances, activated NK cells kill immature DC, favoring the selection of immunogenic DC necessary for an appropriate immune response (17, 18). In addition, DC stimulate NK cell activation while NK cells enhance macrophage activation and promote DC maturation, and NKp30 and cytokines have been involved in both events (19, 20). Therefore, NK cells skew the adaptive immune response toward a T helper (Th) 1 and cytotoxic T lymphocyte (CTL) profiles, both essential for an effective antitumor and antiviral immune response (21–24). Also, the crosstalk between DC and NK cells has been involved in the promotion and protection of autoimmune conditions (25–27).

Interleukin-12 plays a major role in NK cell activation in response to pathogens and tumors (28, 29). It belongs to an extended family of cytokines that share cytokine and receptor subunits and display overlapping functions (30, 31). Recently, we demonstrated that IL-27, another member of IL-12 family, induces human NK cell-mediated cytotoxicity, IFN-γ production, and potentiates antibody-dependent NK cell-mediated cytotoxicity (ADCC) (32). In addition, IL-27 synergizes with IL-18 for the stimulation of NK cell effector functions (32–34). IL-23 is another member of the IL-12 family composed by the IL-12p40 and p19 subunits that signals through a heterodimeric receptor composed by the IL-12Rβ1 and the IL-23R chains (35, 36). Similarly to IL-12 and IL-27, IL-23 is secreted by monocytes, macrophages, and DC in response to bacterial, viral, and fungal components (37, 38). IL-23 increases IFN-γ production by human CD4^+^ T cells (35, 39), and it is also involved in survival, expansion, and activation of Th17 cells (31). Data obtained in mice indicate that IL-23 can both inhibit (40) and activate (41, 42) NK cell responses. However, IL-23 effects on human NK cells remain ill-defined. Therefore, in this work, we explored the role of IL-23 on human NK cell effector functions and demonstrated that it stimulated IFN-γ secretion by CD56^bright^, but not CD56^dim^ cells, primed NK cells for IL-18-driven IFN-γ production, and that NK cells co-stimulated with IL-23 and IL-18 enhanced IL-12 secretion and CD86 expression on DC in an IFN-γ-dependent manner.

## Materials and Methods

### Antibodies and Reagents

Human rIL-2 and rIL-15 (PeproTech), rIL-18 (MBL International), rIL-23 (eBioscience), rGM-CSF (Sigma), and rIL-4 (R&D) were used. Cells were incubated with fluorochrome-coupled mAb against the following human molecules: CD25 (BC96), CD1a (HI149), NKp46 (9E2), NKp30 (P30-15), NKp44 (P44-8), NKp80 (5D12), IFN-γ (4S.B3), T-bet (4B10), CD178 (FasL, NOK-1), CD14 (HCD14), CD83 (HB15e), CD16 (3G8), unlabeled anti-IFN-γ (NIB42), fluorochrome-labeled, and unlabeled isotype-matched controls (IC) from Biolegend; CD226 (DNAM-1, DX11), CD69 (FN50) and CD154 (CD40L, TRAP1) from BD Pharmingen; CD56 (N901) from Beckman Coulter; NKG2C (134591), TRAIL (71908), TIGIT (741182), CD85j (ILT2, 292305), IL-18Rα (H44), IL-18Rβ (132029), and IL-23R (218213) from R&D; CD3 (UCHT-1), HLA-DR (L243), and CD86 (IT2.2) from Tonbo; Eomes (WD1928) from eBioscience; unlabeled anti-IL-23p19 (B-Z23) from Abcam. The following reagents were used at the indicated concentrations: the inhibitor of c-Jun N-terminal kinase (JNK) SP600125 (20 µM, Calbiochem); the Janus kinase 2 (Jak2) inhibitor AG490 (25 µM, Calbiochem); the p38 MAP kinase inhibitor SB202190 (10 µM, Calbiochem); the inhibitor of phosphoinositide 3-kinase (PI3K) Ly294002 (2 µM, Sigma); the inhibitor of MEK1/MEK2 kinases (MAPKs) U0126 (5 µM, Sigma); the inhibitor of cytokine-induced IκBα phosphorylation BAY11-7082 (1 µM, Sigma); the inhibitor of the mammalian target of rapamycin (mTOR) rapamycin (5 nM, Sigma); the inhibitor of STAT1 Fludarabine (0.1 µg/ml, Fludara^®^ Schering). LPS (*E. coli* 0111:B4 strain, Sigma) was used at 0.1 or 1 µg/ml. The dose of each pharmacological inhibitor used in the experiments was established in previous work (13, 32) and did not affect NK cell viability. Rituximab (RTX, Roche) and normal human polyclonal IgG (IgG2500, Purissimus, Argentina) were used at 10 µg/ml.

### Monocytes, DC, and NK Cells

Buffy coats from healthy volunteers were provided by the Blood Bank of the “Carlos Durand” Hospital or by the “Complejo Médico Churruca-Visca” (Buenos Aires, Argentina). Monocytes (CD14^+^ cells) were isolated by MACS (Miltenyi); NK cells were isolated using RosetteSep (StemCell) and Ficoll-Paque™ Plus (GE Life Sciences) centrifugation. Purity of isolated cells was always above 90%, as assessed by flow cytometry (FC; CD14^+^ cells or CD3^−^CD56^+^). Monocytes (1 × 10^5^) were incubated for 24 h with LPS (1 µg/ml), then, NK cells (1 × 10^5^) were added for another 24 h in the presence of an IC mAb or a neutralizing anti-IL-23p19 mAb (10 µg/ml) and IFN-γ was evaluated in the supernatants. Also, cell culture supernatants of monocytes incubated for 48 h with LPS were used for analysis of IL-23 production. Monocytes were cultured for 6 days with GM-CSF and IL-4 to obtain immature DC (iDC) characterized as CD1a^+^MHC-II^low^CD83^−/low^CD86^−/low^. DCs (1 × 10^5^) were cultured for 18 h with previously stimulated and washed NK cells (1 × 10^5^) plus LPS (0.1 µg/ml) in the absence or in the presence of an IC mAb or a neutralizing anti-IFN-γ mAb (10 µg/ml). Cells were cultured in RPMI 1640 (Gibco) supplemented with 10% inactivated fetal bovine serum (Gibco), sodium pyruvate, glutamine, and gentamicin (Sigma). Cell culture supernatants were collected and used for analysis of IL-12 production and cells were used to assess CD86 expression. Also, NK cells (1 × 10^6^/ml) were cultured for 24 h or 5 days in the presence of IL-15 (4 ng/ml) and in the absence or in the presence of IL-18 (10 ng/ml), IL-23 (10 ng/ml), or their combination. In some experiments, NK cells stimulated with IL-23 were thereafter stimulated with IL-2 (8 ng/ml) for 24 h. For dose–response experiments, IL-23 was also used at 1 ng/ml. Cells were used for phenotypic analysis, cytokine production, proliferation, and cell death evaluation. For cytotoxicity assessment and NK cell “helper” function evaluation, NK cells (2 × 10^6^/ml) were incubated overnight. To investigate the signaling pathways involved in the IFN-γ response, NK cells were incubated with pharmacologic inhibitors for 45–60 min and then stimulated with the different cytokines for another 23 h. For priming experiments, NK cells (1 × 10^6^/ml) were cultured overnight in the absence or in the presence of IL-18 or IL-23, extensively washed and further cultured (1 × 10^6^/ml) for 24 h in the absence or in the presence of IL-23 or IL-18, as indicated in the figure. Studies have been approved by the institutional review committee and informed consent of participating subjects was obtained.

### NK Cell Proliferation

Natural killer cells cultured for 5 days as described, were pulsed with 1 μCi/well of methyl-3H-thymidine (3H-Thy; New England Nuclear Life Science) during the last 18 h of cell culture, harvested on glass-fiber filters, and incorporated radioactivity was measured in a liquid scintillation counter. Results are expressed as mean counts per minute (cpm) of triplicate wells ± SEM.

### Flow Cytometry and Cell Sorting

Expression of cell surface receptors on NK cells or DC was analyzed by FC as previously described (43). Expression of IFN-γ, Eomes and T-bet was analyzed by intracellular FC using Cytofix/Cytoperm (BD). For assessment of IFN-γ production, cells were cultured in the presence of Golgi-Plug^®^ and Golgi-Stop^®^ reagents during the last 4 h. For CD40L analysis, cells were cultured in the presence of the specific mAb and Golgi-Stop^®^ during the last 6 h, as described (44) with slight modifications. Human CD40L-transfected fibroblasts were used as positive controls. Samples were acquired in a FACSCanto II-plus flow cytometer (BD) or MACSQuant Analyzer 10 (Miltenyi Biotec). Data were analyzed using FlowJo software (Tree Star). Results were expressed as percentage of positive cells or MFI. For cell sorting, NK cells were isolated using RosetteSep (StemCell), stained with mAb against CD56 and CD16 and then CD56^bright^ (CD56^high^CD16^−/low^) and CD56^dim^ (CD56^dim^CD16^high^) NK cells were sorted in a FACSAria II-plus cell sorter (BD Biosciences).

### ELISA

Secretion of IFN-γ (Biolegend), IL-12 (ELISA MAX Standard kit, Biolegend), and IL-23 (DuoSet, R&D) was analyzed by ELISA as described (13).

### Cytokine Bead Array

Secretion of IL-4, IL-10, IL-17, IL-6, TNF, and IFN-γ by cytokine-stimulated NK cells was analyzed by the CBA (BD) as indicated by the manufacturer.

### NK Cell-Mediated Cytotoxicity

Natural killer cells were cultured overnight with IL-15 in the absence or in the presence of IL-18, IL-23, or the combination of both, washed, and co-cultured for 5 h with CFSE-labeled Raji (*Burkitt’s lymphoma*, ATCC) at different E:T ratios. Cells were thereafter labeled with 7-AAD and analyzed by FC. Percentage of cytotoxicity was calculated as 100 × percentage of CFSE^+^7-AAD^+^ cells/percentage of CFSE^+^ cells. Percentage of spontaneous dead cells (without effector NK cells) was always below 5%. For ADCC, CFSE-labeled Raji cells previously incubated with RTX or normal human IgG for 2 h were used as target at a 1:1 E:T ratio.

### Statistical Analysis

Paired *t*-test or Wilcoxon matched paired test (when data did not pass the normality test) were used when two experimental groups were compared. A one-way ANOVA test with Bonferroni *post hoc* test was used when three or more experimental groups were compared. A one-way ANOVA test with Dunnett’s *post hoc* test was used in pharmacologic inhibition experiments. When data did not pass normality test, Friedman test with Dunn’s *post hoc* test was used instead. A two-way ANOVA with repeated measures and Bonferroni *post hoc* test was used for IFN-γ production by sorted NK cells and by IL-23 plus IL-18-stimulated NK cells, for cytotoxicity and for DC-NK cell co-cultures in the presence of neutralizing mAb experiments. The interaction effect in the 2 × 2 factorial ANOVA was performed for the definition of synergism, and *P* values for it were reported in the legends of the figures (45). Data were analyzed using GraphPad Prism 6.0 software.

## Results

### Monocytes Produce IL-23 That Stimulates NK Cell IFN-γ Production

**Figure 1:**
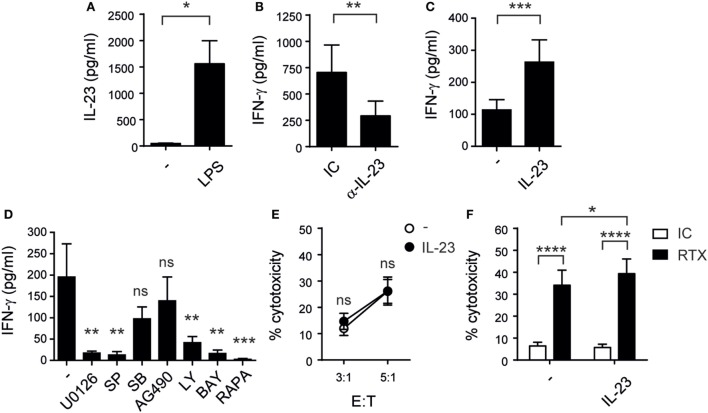
Monocyte-derived interleukin (IL)-23 promotes IFN-γ secretion by human natural killer (NK) cells while cytotoxicity remains unaltered after rIL-23 stimulation. **(A)** IL-23 secretion by monocytes cultured for 48 h in the absence (−) or in the presence of LPS; *n* = 8. **(B)** IFN-γ secretion by NK cells upon coculture for 24 h with monocytes pre-stimulated for 24 h with LPS, in the presence of an IC mAb (IC) or a blocking mAb against IL-23 (α-IL-23); *n* = 9. **(C)** IFN-γ secretion by resting NK cells cultured for 24 h in the absence (−) or in the presence of 10 ng/ml of IL-23; *n* = 23. **(D)** IFN-γ secretion by NK cells stimulated for 24 h with 10 ng/ml of IL-23 in the absence (−) or in the presence of U0126, SP600125 (SP), SB202190 (SB), AG490, Ly294002 (Ly), BAY11-7082 (BAY), or rapamycin (RAPA); *n* = 6. **(E)** Cytotoxic activity of NK cells previously cultured overnight in the absence (○) or in the presence (●) of IL-23 against Raji cells at different E:T ratios; *n* ≥ 6. **(F)** ADCC of NK cells previously cultured in the absence (−) or in the presence of IL-23 against Raji cells incubated with an IC mAb (white bars) or against RTX-coated Raji cells (black bars) at 1:1 E:T ratio; *n* = 6. Mean ± SEM are shown. ns, not significant; * *p* < 0.05; **p < 0.01; ***p < 0.001; ****p < 0.0001; paired *t*-test **(A)**, Wilcoxon test **(B,C)**, one-way ANOVA with Dunnett’s *post hoc* test **(D)**, and two-way ANOVA with Bonferroni’s *post hoc* test **(E,F)**.

To assess whether IL-23 participates in the crosstalk between monocytes and NK cells, we first evaluated the capacity of monocytes to produce this cytokine. Accordingly, we observed that they secreted IL-23 upon stimulation with LPS (Figure 1A). Moreover, IL-23 blockade led to a significant reduction in the amounts of IFN-γ secreted by NK cells during their coculture with LPS-stimulated monocytes (Figure 1B). Next, we stimulated isolated NK cells with recombinant human IL-23 and confirmed that this cytokine-induced IFN-γ production not only by resting (Figure 1C) but also by NK cells previously activated with a combination of IL-12, IL-15, and IL-18 (data not shown). Conversely, IL-23 did not promote the secretion of IL-4, IL-10, IL-17, IL-6, and TNF by NK cells, assessed by Cytokine Bead Array (CBA) and FC (data not shown). Pharmacologic inhibition revealed that MEK1/MEK2, JNK, PI3K, mTOR, and NF-κB but not p38 MAPK, Jak2 (Figure 1D), or STAT1 (data not shown) were involved in the IL-23-driven IFN-γ response. Besides, IL-23 did not affect NK cell-mediated cytotoxicity (Figure 1E). Accordingly, this cytokine did not affect the expression of the activating receptors CD335 (NKp46), CD336 (NKp44), CD337 (NKp30), NKp80 (KLRF1), CD226 (DNAM-1), NKG2C nor the expression of TRAIL and CD178/FasL (data not shown). Therapeutic efficacy of humanized monoclonal antibodies directed against tumor cell surface-expressed molecules relies partially on ADCC. We observed that RTX-coated Raji cells were susceptible to NK cell-mediated cytotoxicity and that IL-23 further increased such susceptibility in a statistically significant manner (Figure 1F). Of note, IL-23 did not alter the expression levels of CD16 on NK cells (data not shown).

**Figure 2:**
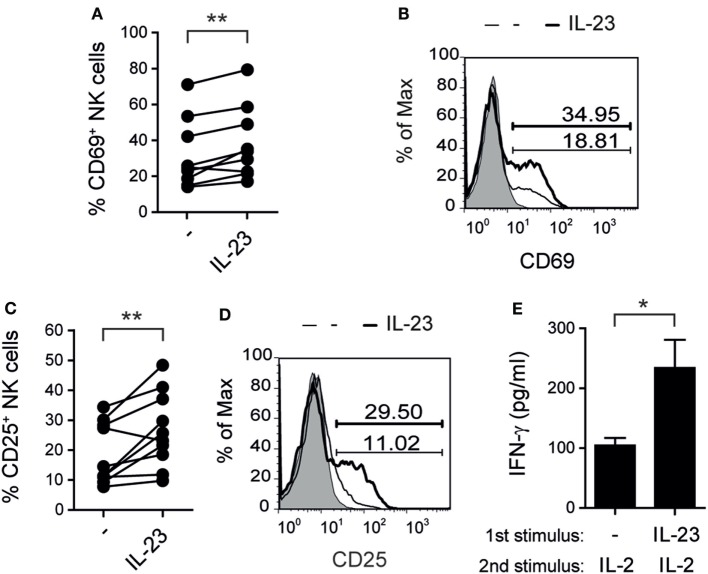
Interleukin (IL)-23 activates natural killer (NK) cells. **(A–D)** Percentage of NK cells expressing CD69 **(A,B)** or CD25 **(C,D)** after 5 days of culture in the absence (−) or in the presence of 10 ng/ml of IL-23; *n* = 9 **(A)** and *n* = 10 **(C)**. Representative histograms are shown in **(B,D)**. Gray: IC. Thin line: unstimulated NK cells. Thick line: NK cells stimulated with IL-23. Numbers within histograms: percentage of positive cells for each marker in each condition. **(E)** IFN-γ secretion by NK cells cultured for 5 days in the absence (−) or in the presence of 10 ng/ml of IL-23 and thereafter re-stimulated 24 h with 8 ng/ml of IL-2; *n* = 7. Mean ± SEM are shown in **(E)**. ns, not significant; *p < 0.05; **p < 0.01; paired *t*-test **(A,C,E)**.

In addition, IL-23 induced a higher percentage of CD69^+^ (Figures 2A,B) and CD25^+^ (Figures 2C,D) NK cells indicating that this cytokine promoted NK cell activation. Furthermore, NK cells first incubated with IL-23 secreted higher amounts of IFN-γ than non-stimulated NK cells when they were re-stimulated with IL-2, suggesting that the effect of IL-23 on CD25 expression had functional consequences (Figure 2E).

**Figure 3:**
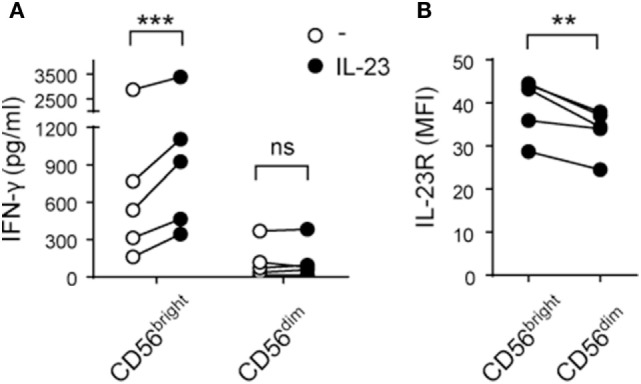
CD56^bright^ natural killer (NK) cells secrete IFN-γ in response to interleukin (IL)-23 and express higher amounts of IL-23R than CD56^dim^ NK cells. **(A)** IFN-γ secretion by FACS-sorted CD56^bright^ and CD56^dim^ NK cells cultured for 24 h in the absence (○) or in the presence (●) of IL-23; *n* = 5. **(B)** IL-23 receptor (IL-23R) expression on CD56^bright^ and CD56^dim^ NK cells; *n* = 5. ns, not significant; **p < 0.01; ***p < 0.001; two-way ANOVA with Bonferroni’s *post hoc* test **(A)** and paired *t*-test **(B)**.

Next, we analyzed intracellular IFN-γ production by CD56^bright^ and CD56^dim^ NK cells in response to IL-23. Although we did not detect differences in the percentage of IFN-γ^+^ NK cells in these subpopulations, we observed higher amounts of IFN-γ expression in CD56^bright^ but not in CD56^dim^ NK cells stimulated with IL-23 compared to unstimulated cells (data not shown). To confirm this result, we FACS-sorted CD56^bright^ and CD56^dim^ NK cells and observed a statistically significant increase in IFN-γ secretion by CD56^bright^ but not CD56^dim^ NK cells upon IL-23 stimulation (Figure 3A). Accordingly, CD56^bright^ NK cells expressed higher amounts of IL-23 receptor (IL-23R) than CD56^dim^ NK cells (Figure 3B).

### IL-23 Primes NK Cells for IL-18-Induced IFN-γ Production

**Figure 4:**
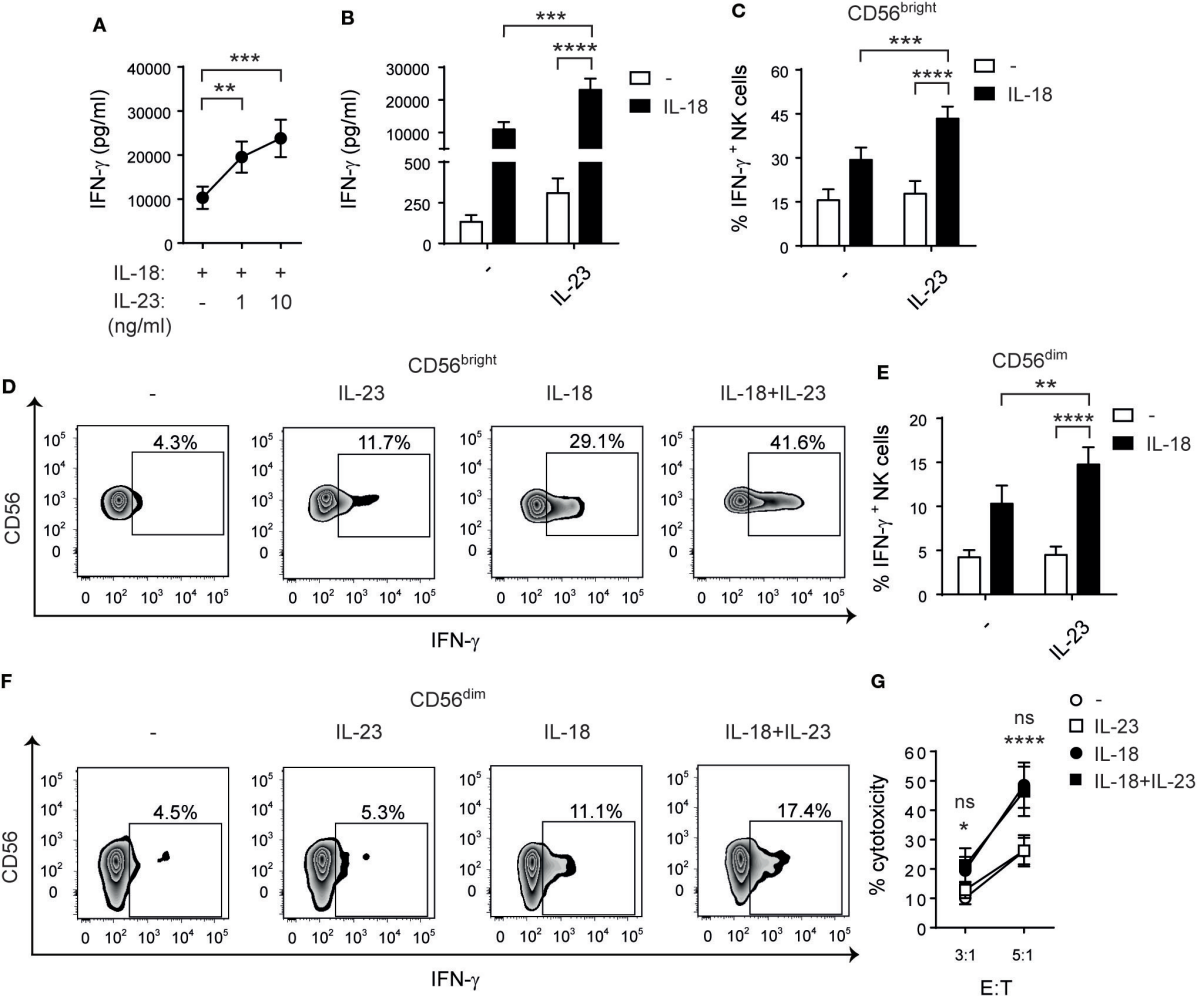
Interleukin (IL)-23 synergizes with IL-18 for IFN-γ production but not for cytotoxicity. **(A)** IFN-γ secretion by natural killer (NK) cells cultured for 24 h with IL-18 in the absence (−) or in the presence of 1 or 10 ng/ml of IL-23; *n* = 14. **(B)** IFN-γ secretion by NK cells cultured for 24 h in the absence (−) or in the presence of IL-23, IL-18, or IL-23 and IL-18 (IL-18 + IL-23), all at 10 ng/ml; *n* = 17. **(C–F)** Percentage of IFN-γ producing CD56^bright^ **(C,D)** and CD56^dim^ **(E,F)** NK cells after culture in the absence (−) or in the presence of IL-23, IL-18, or IL-23 and IL-18 for 24 h; *n* = 12. Representative zebra plots are shown in **(D,F)**. **(G)** Cytotoxic activity of NK cells previously cultured overnight in the absence (−) or in the presence of IL-23, IL-18, or IL-23 and IL-18 against Raji cells at different E:T ratios; *n* ≥ 6. Mean ± SEM are shown. ns, not significant; *p < 0.05; **p < 0.01; ***p < 0.001; ****p < 0.0001; one-way ANOVA with repeated measures and Bonferroni’s *post hoc* test **(A)** and two-way ANOVA with repeated measures and Bonferroni’s *post hoc* test **(B,C,E,G)**. The interaction *p* values (synergism) were: *p* = 0.0038 **(B)**, *p* = 0.0053 **(C)**, and *p* = 0.0110 **(E)**. **(G)** *NK cells stimulated with IL-18 + IL-23 vs IL-23; ns: IL-18 + IL-23 vs IL-18.

Previous reports demonstrated that IL-12 (46) and IL-27 (32) display a cooperative effect with IL-18 on the activation of NK cell effector functions. Therefore, we investigated whether IL-23 cooperates with IL-18 for NK cell stimulation. First, we performed a dose–response curve and observed a statistically significant increase in the secretion of IFN-γ when NK cells were stimulated with two different concentrations of IL-23 plus IL-18 in comparison with IL-18 alone (Figure 4A). We confirmed the existence of a cooperative effect with a larger number of donors and using resting (Figure 4B) and activated NK cells (data not shown). Statistical analysis based on testing the interaction effect in a two-way ANOVA (45) demonstrated the existence of a synergistic effect between IL-23 and IL-18. Moreover, in opposition to the effect of IL-23 alone, we detected that this synergistic effect was noticeable both on CD56^bright^ (Figures 4C,D) and on CD56^dim^ (Figures 4E,F) NK cells. As IL-23 only exerted an effect on IFN-γ production by CD56^dim^ NK cells when combined with IL-18, we explored whether such combination also affected NK cell-mediated cytotoxicity. However, even in combination with IL-18, IL-23 did not regulate NK cell-mediated cytotoxic activity (Figure 4G).

**Figure 5:**
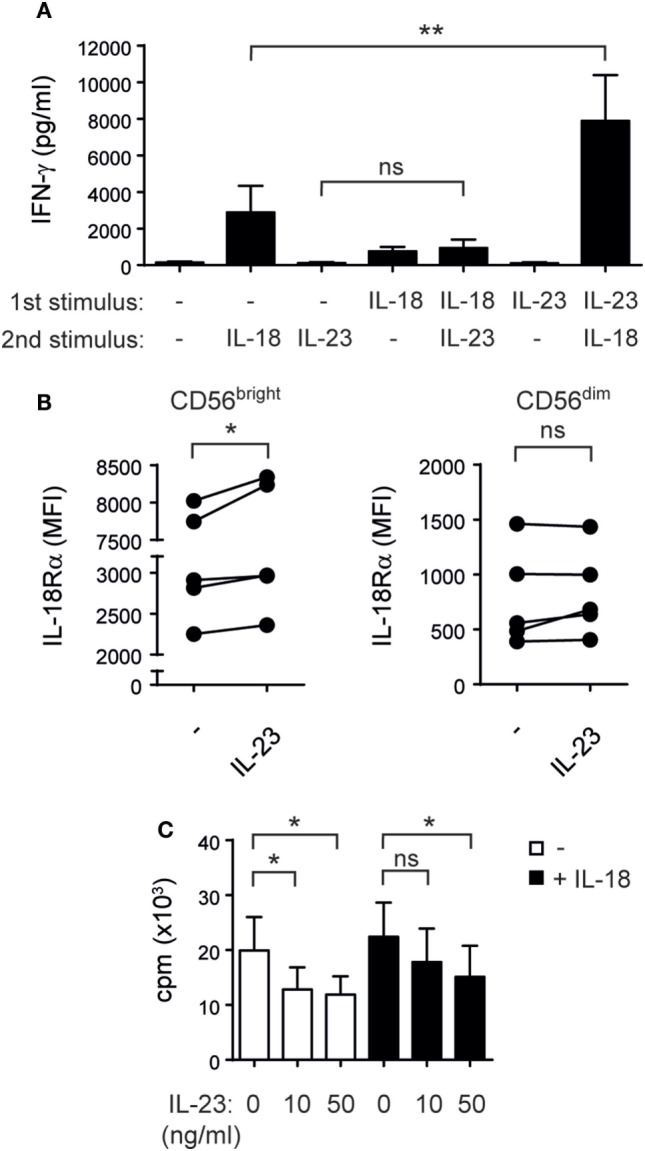
Interleukin (IL)-23 primes natural killer (NK) cells for IL-18-driven IFN-γ secretion upregulating IL-18Rα and inhibits NK cell proliferation. **(A)** IFN-γ secretion by NK cells cultured overnight in the absence (−) or in the presence of IL-23 or IL-18 (“first stimulus”), washed, and thereafter incubated for 24 h in the absence (−) or in the presence of IL-23 or IL-18 (“second stimulus”); *n* = 7. **(B)** IL-18Rα expression on sorted CD56^bright^ and CD56^dim^ NK cells cultured overnight in the absence (−) or in the presence of IL-23; *n* = 5. **(C)** Proliferation of NK cells incubated for 5 days with 1 ng/ml of IL-15 and the indicated doses of IL-23 in the absence (white bars) or in the presence of 10 ng/ml of IL-18 (black bars); *n* = 6. Mean ± SEM are shown. ns, not significant; *p < 0.05; **p < 0.01; one-way ANOVA with repeated measures and Bonferroni’s *post hoc* test.

To further interrogate the cause of the synergistic effect induced by IL-23 and IL-18 on IFN-γ production by NK cells, we performed sequential stimulations. We observed that pre-stimulation of NK cells with IL-18 and subsequent stimulation with IL-23 induced a minor increase in IFN-γ secretion. Conversely, pre-stimulation of NK cells with IL-23 and subsequent stimulation with IL-18 induced a significant increased IFN-γ secretion compared to NK cells not pretreated with IL-23 (Figure 5A). Therefore, the synergistic effect was due to a priming of NK cells by IL-23 for IL-18 responsiveness.

To explore the mechanisms involved in the priming effect, we addressed the expression of T-bet, Eomes, IL-18Rα, and IL-18Rβ in NK cells stimulated with IL-23. While T-bet and IL-18Rβ expression remain unchanged, IL-23 induced a decrease in Eomes expression (data not shown) and a statistically significant upregulation of the expression of IL-18Rα in CD56^bright^ but not CD56^dim^ NK cells (Figure 5B). Therefore, upregulated expression of IL-18Rα induced by IL-23 may explain the increased responsiveness of NK cells to IL-18.

As IL-23 promotes the proliferation of memory T cells and the increased levels of IFN-γ observed could also be a result of an increase in the total number of NK cells, we investigated the effect of IL-23 on NK cells proliferation. Surprisingly, IL-23 exerted a statistically significant inhibition of NK cell proliferation triggered by IL-15 and IL-15 plus IL-18 (Figure 5C) without affecting NK cell viability (data not shown).

### IL-23 Cooperates with IL-18 in the Promotion of DC Activation

**Figure 6:**
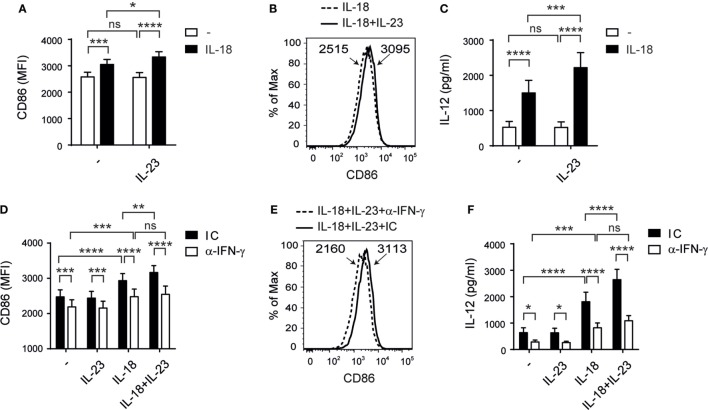
Interleukin (IL)-23 cooperates with IL-18 for natural killer (NK)-driven dendritic cells (DC) activation. **(A–C)** CD86 expression on DC **(A,B)** and IL-12 secretion by DC **(C)** after incubation with LPS and NK cells pre-cultured in the absence (−) or in the presence of IL-23, IL-18, or IL-23 and IL-18 (IL-18 + IL-23); *n* = 11. Representative histograms of CD86 expression on DC cultured with NK cells pre-stimulated with IL-18 or IL-18 and IL-23 (IL-18 + IL-23) are shown in **(B)**. **(D–F)** CD86 expression on DC **(D,E)** and IL-12 secretion by DC **(F)** after incubation with LPS and NK cells pre-cultured in the absence (−) or in the presence of IL-23, IL-18, or IL-23 and IL-18, in the presence of an IC mAb (black bars) or an anti-IFN-γ neutralizing mAb (white bars); *n* = 9. Representative histograms of CD86 expression on DC cultured with NK cells pre-stimulated with IL-18 and IL-23, in the presence of an IC mAb or an anti-IFN-γ neutralizing mAb are shown in **(E)**. Numbers within histograms represent the MFI of CD86 in each condition. Data are shown as mean ± SEM in panels **(A,C,D,F)**. ns, no significant; *p < 0.05; **p < 0.01; ***p < 0.001; ****p < 0.0001; two-way ANOVA with repeated measures matched by both factors and Bonferroni’s *post hoc* test **(A,C,D,F)**. The interaction *p*-values (synergism) were: *p* = 0.0287 **(A)** and *p* = 0.0028 **(C)**.

As NK cell–DC crosstalk shapes adaptive immunity, we examined whether NK cells co-stimulated with IL-23 and IL-18 affect DC maturation. NK cells were stimulated overnight in the absence or in the presence of IL-23, IL-18, or both, and then cocultured with DC pulsed with LPS. We observed a statistically significant increase in CD86 expression (Figures 6A,B) but not in CD83 or HLA-DR expression (data not shown) on DC when NK cells were pre-stimulated with IL-23 and IL-18 compared to DC cocultured with NK cells stimulated with each cytokine alone. Moreover, we observed an increased secretion of IL-12 (Figure 6C) by DC when NK cells were pre-stimulated with both cytokines. To investigate the underlying mechanisms, we stimulated NK cells with IL-23, IL-18, or IL-23 and IL-18, and we evaluated the expression of CD40L, DNAM-1, NKG2D, NKG2C, NKp30, NKp46, TIGIT, and ILT2 on NK cells. However, we did not observe differences that could suggest the involvement of any of these receptors in the potentiation of DC activation when they were cocultured with NK cells pre-stimulated with IL-23 and IL-18 (data not shown). Conversely, neutralization of IFN-γ during the cocultures of DC and NK cells pre-stimulated with the combination of the cytokines abrogated both effects, CD86 expression (Figures 6D,E), and IL-12 secretion (Figure 6F). Therefore, IL-23 in concert with IL-18 promotes NK cell activation that drives DC maturation mainly by IFN-γ produced by NK cells.

## Discussion

Interleukin-23 has been involved in immunity against tumors (29, 47) and acute infections (30, 31) but also contributes to tumor promotion and growth (40, 48) and the development of autoimmune diseases, mainly through the induction of a Th17 response (30, 49). These opposing functions may depend on the context and/or on the immune cells on which IL-23 exerts its effects. In mice, IL-23 triggers pro- (40) and antitumoral (41, 42) effects on NK cells. However, little is known about its effect on human NK cells (50). In this study, we demonstrated that monocytes incubated with LPS secrete IL-23 that stimulates IFN-γ secretion mostly by CD56^bright^ NK cells, which could be due to the observation that CD56^bright^ NK cells express higher amounts of IL-23R than CD56^dim^ NK cells. Of note, we used low doses of IL-15 as survival factor and to prime NK cells as it has been shown that resting NK cells require at least two signals for efficient IFN-γ secretion (9, 12, 21, 51, 52). Also, as the secretion of cytokines is usually restricted to the synaptic cleft between DC and NK cells (53, 54), the concentration of cytokines in sera might be quite different from the concentration that NK cells sense. Nevertheless, the concentration of IL-23 used in our experiments is within the range of values detected in sera from patients with different physiopathological conditions (55, 56). Besides, they are similar to the concentration of IL-23 detected in supernatants from monocyte-derived DCs of multiple sclerosis patients stimulated with LPS (57) or produced by macrophages and DC stimulated with pathogens or TLR ligands (37, 58, 59).

In addition, in this study, we observed that IL-23-driven IFN-γ production requires functional MEK1/MEK2, JNK, PI3K, mTOR, and NF-κB, but not STAT-1, STAT-3 (a downstream mediator of Jak2), nor p38 MAPK. All these signaling pathways have previously been implicated in NK cell effector functions (13, 32, 60, 61).

Interleukin-4, IL-10, IL-17, and TNF were shown to be secreted by NK cells under certain conditions and different immune cells stimulated with IL-23 produce IL-17, IL-6, and TNF (30, 62–65). However, IL-23 did not affect their secretion by NK cells. Nevertheless, we observed a raise in the percentage of CD69^+^ and CD25^+^ NK cells upon stimulation with IL-23, confirming that this cytokine activates NK cells. Moreover, IL-23 potentiated ADCC (mediated through CD16) but not NK cell-mediated cytotoxicity through other NK cell activating receptors, suggesting that this cytokine may act as a co-stimulus not only for IFN-γ production but also for CD16-mediated cytotoxicity. Interestingly, potentiation of ADCC is a biological effect that IL-23 shares with IL-12 and IL-27 (32, 60), which suggests that these cytokines of the IL-12 family of cytokines might be suitable candidates as adjuvant therapy during immunotherapy with humanized mAb.

Myeloid cells can produce IL-18 upon recognition of tumors and pathogens, which in combination with other stimuli such as IL-12 or IL-27 activates NK cells (32, 34, 46). Here, we demonstrated that IL-23, like the other members of the same family of cytokines, displays a synergistic effect with IL-18 for NK cell-mediated IFN-γ production by both CD56^bright^ and CD56^dim^ cells. Interestingly, this effect is due to a priming of IL-23 for IL-18-responsiveness and not to a proliferative effect on NK cells or to changes in their viability. On the contrary, we observed an IL-23-driven inhibition of NK cell proliferation similar to what we have already seen for another member of the IL-12 family of cytokines, IL-27 (32). The upregulation of IL-18Rα expression on CD56^bright^ NK cells observed in our experiments might be responsible for the priming effect that led to an increased responsiveness of NK cells to IL-18 for IFN-γ production.

Remarkably, CD56^dim^ NK cells only produced IFN-γ in response to the simultaneous stimulation with both cytokines but not in response to IL-23 alone, as did CD56^bright^ NK cells. However, NK cell-mediated cytotoxicity was not affected when NK cells were stimulated with IL-23 or with IL-23 and IL-18. Therefore, IL-23 might display a predominant immunoregulatory but not a cytotoxicity-inducing effect on NK cells, which is in contrast with the effect of other members of the same family of cytokines such as IL-12 and IL-27 (28, 29, 32). These results further support the notion that both effector functions are differentially regulated in NK cells (13, 66). Also, the lack of effect of IL-23 on NK cell cytotoxicity could be due to the fact that CD56^dim^ NK cells express less IL-23R than CD56^bright^ NK cells. Nonetheless, IL-23 induced IFN-γ secretion by CD56^dim^ NK cells in combination with IL-18 and enhanced ADCC, which indicates that it may act as co-stimulatory cytokine when acting in concert with another primary stimulus for NK cells.

Immature DC are mainly localized in peripheral tissues and migrate to secondary lymphoid organs upon maturation induced by PAMP or tumors to induce an adaptive immune response. NK cells activated upon target cell recognition or by cytokines such as IL-2 or IL-12 induce CD86 expression and IL-12 production by DCs (15, 21, 24, 67). Therefore, we hypothesized that IL-23 and IL-18 secreted by myeloid cells during the onset of an immune response may activate NK cells that in turn might affect maturation of DC that already sensed PAMP, which in turn may affect T cell priming. To mimic this situation *in vitro* with human cells, we used LPS during DC–NK cell cocultures to activate DC. We observed that NK cells pre-stimulated with IL-18 induced heightened secretion of IL-12 and increased expression of CD86 on DC activated with LPS, and that this effect is further potentiated by IL-23. This effect could be related to the “helper” function acquired by NK cells exposed to IL-18 that leads to the stimulation of IL-12 production by DC activated through CD40 and the development of a Th1 response (51). In our experimental setting, we did not observe differences in the expression of CD40L as other authors did (68, 69), or other activating or inhibitory receptors on NK cells that could suggest the involvement of surface molecules in the potentiation of DC activation. Conversely, we observed that IL-23 enhanced IL-18-driven NK cell “helper” function mainly through IFN-γ production.

Our results show that the effects of IL-23 on NK cells are mostly immunoregulatory and that this cytokine cooperates with IL-18, which suggest that IL-23 may actually functions as a co-stimulatory cytokine that principally potentiates the effect of IL-18 in NK cells. These observations also explain the pro- or antitumoral effects described for IL-23 on NK cells (29, 40–42, 47, 48), which may critically depend on the composition of the tumor microenvironment, in particular, the presence of other cytokines that regulate NK cell effector functions.

Overall, we conclude that IL-23 produced by activated myeloid cells induces NK cell activation and IFN-γ secretion, which enhances IL-18-driven NK cell “helper” activity that fosters DC maturation and IL-12 secretion. Therefore, our results unravel the stimulatory functions of IL-23 on NK cells, and their possible impact on the Th1- and CTL-mediated adaptive immune response against tumors and pathogens.

## Ethics Statement

Studies have been approved by the institutional review committee from IBYME.

## Author Contributions

AZ participated in the design of the study, performed and analyzed the experiments, prepared the figures, and wrote and corrected the manuscript. SN, XI, RS, FS, NT, JS, CD, and MF assisted in performing experiments and analyzing results. NZ designed the study, analyzed the experiments, and corrected the manuscript. All authors reviewed the results and approved the final version of the manuscript.

## Conflict of Interest Statement

The authors declare that the research was conducted in the absence of any commercial or financial relationships that could be construed as a potential conflict of interest.

## References

[1] Artis D Spits H. The biology of innate lymphoid cells. Nature (2015) 517:293–301. 10.1038/nature14189 25592534

[2] Lodoen MB Lanier LL. Natural killer cells as an initial defense against pathogens. Curr Opin Immunol (2006) 18:391–8. 10.1016/j.coi.2006.05.002 16765573 PMC7127478

[3] Vivier E Tomasello E Baratin M Walzer T Ugolini S. Functions of natural killer cells. Nat Immunol (2008) 9:503–10. 10.1038/ni1582 18425107

[4] Cooper MA Fehniger TA Caligiuri MA. The biology of human natural killer-cell subsets. Trends Immunol (2001) 22:633–40. 10.1016/S1471-4906(01)02060-9 11698225

[5] Fauriat C Long EO Ljunggren HG Bryceson YT. Regulation of human NK-cell cytokine and chemokine production by target cell recognition. Blood (2010) 115:2167–76. 10.1182/blood-2009-08-238469 19965656 PMC2844017

[6] De Maria A Bozzano F Cantoni C Moretta L Revisiting human natural killer cell subset function revealed cytolytic CD56(dim)CD16+ NK cells as rapid producers of abundant IFN-gamma on activation. Proc Natl Acad Sci U S A (2011) 108:728–32. 10.1073/pnas.1012356108 21187373 PMC3021076

[7] Di Santo JP. Functionally distinct NK-cell subsets: developmental origins and biological implications. Eur J Immunol (2008) 38:2948–51. 10.1002/eji.200838830 18979515

[8] Caligiuri MA. Human natural killer cells. Blood (2008) 112:461–9. 10.1182/blood-2007-09-077438 18650461 PMC2481557

[9] Ferlazzo G Pack M Thomas D Paludan C Schmid D Strowig T Distinct roles of IL-12 and IL-15 in human natural killer cell activation by dendritic cells from secondary lymphoid organs. Proc Natl Acad Sci U S A (2004) 101:16606–11. 10.1073/pnas.0407522101 15536127 PMC534504

[10] Lanier LL NK cell recognition. Annu Rev Immunol (2005) 23:225–74. 10.1146/annurev.immunol.23.021704.115526 15771571

[11] Zwirner NW Domaica CI. Cytokine regulation of natural killer cell effector functions. Biofactors (2010) 36:274–88. 10.1002/biof.107 20623510

[12] Fehniger TA Shah MH Turner MJ VanDeusen JB Whitman SP Cooper MA Differential cytokine and chemokine gene expression by human NK cells following activation with IL-18 or IL-15 in combination with IL-12: implications for the innate immune response. J Immunol (1999) 162:4511–20. 10201989

[13] Girart MV Fuertes MB Domaica CI Rossi LE Zwirner NW Engagement of TLR3, TLR7, and NKG2D regulate IFN-gamma secretion but not NKG2D-mediated cytotoxicity by human NK cells stimulated with suboptimal doses of IL-12. J Immunol (2007) 179:3472–9. 10.4049/jimmunol.179.6.3472 17804388

[14] Michel T Hentges F Zimmer J Consequences of the crosstalk between monocytes/macrophages and natural killer cells. Front Immunol (2012) 3:403 10.3389/fimmu.2012.00403 23316194 PMC3539656

[15] Walzer T Dalod M Robbins SH Zitvogel L Vivier E Natural-killer cells and dendritic cells: “l’union fait la force”. Blood (2005) 106:2252–8. 10.1182/blood-2005-03-1154 15933055

[16] Chijioke O Munz C Dendritic cell derived cytokines in human natural killer cell differentiation and activation. Front Immunol (2013) 4:365 10.3389/fimmu.2013.00365 24273539 PMC3822368

[17] Wilson JL Heffler LC Charo J Scheynius A Bejarano MT Ljunggren HG Targeting of human dendritic cells by autologous NK cells. J Immunol (1999) 163:6365–70. 10586025

[18] Ferlazzo G Moretta L. Dendritic cell editing by natural killer cells. Crit Rev Oncog (2014) 19:67–75. 10.1615/CritRevOncog.2014010827 24941374

[19] Ferlazzo G Tsang ML Moretta L Melioli G Steinman RM Münz C. Human dendritic cells activate resting natural killer (NK) cells and are recognized via the NKp30 receptor by activated NK cells. J Exp Med (2002) 195:343–51. 10.1084/jem.20011149 11828009 PMC2193591

[20] Vitale M Della Chiesa M Carlomagno S Pende D Aricò M Moretta L NK-dependent DC maturation is mediated by TNFalpha and IFNgamma released upon engagement of the NKp30 triggering receptor. Blood (2005) 106:566–71. 10.1182/blood-2004-10-4035 15784725

[21] Mailliard RB Son YI Redlinger R Coates PT Giermasz A Morel PA Dendritic cells mediate NK cell help for Th1 and CTL responses: two-signal requirement for the induction of NK cell helper function. J Immunol (2003) 171:2366–73. 10.4049/jimmunol.171.5.2366 12928383

[22] Martin-Fontecha A Thomsen LL Brett S Gerard C Lipp M Lanzavecchia A Induced recruitment of NK cells to lymph nodes provides IFN-gamma for T(H)1 priming. Nat Immunol (2004) 5:1260–5. 10.1038/ni1138 15531883

[23] Boehm U Klamp T Groot M Howard JC Cellular responses to interferon-gamma. Annu Rev Immunol (1997) 15:749–95. 10.1146/annurev.immunol.15.1.749 9143706

[24] Gerosa F Baldani-Guerra B Nisii C Marchesini V Carra G Trinchieri G. Reciprocal activating interaction between natural killer cells and dendritic cells. J Exp Med (2002) 195:327–33. 10.1084/jem.20010938 11828007 PMC2193595

[25] Terrazzano G Sica M Gianfrani C Mazzarella G Maurano F De Giulio B Gliadin regulates the NK-dendritic cell cross-talk by HLA-E surface stabilization. J Immunol (2007) 179:372–81. 10.4049/jimmunol.179.1.372 17579058

[26] Chong WP van Panhuys N Chen J Silver PB Jittayasothorn Y Mattapallil MJ NK-DC crosstalk controls the autopathogenic Th17 response through an innate IFN-gamma-IL-27 axis. J Exp Med (2015) 212:1739–52. 10.1084/jem.20141678 26347474 PMC4577839

[27] Gianchecchi E Delfino DV Fierabracci A. NK cells in autoimmune diseases: linking innate and adaptive immune responses. Autoimmun Rev (2017). 10.1016/j.autrev.2017.11.018 29180124

[28] Scharton-Kersten T Afonso LC Wysocka M Trinchieri G Scott P. IL-12 is required for natural killer cell activation and subsequent T helper 1 cell development in experimental leishmaniasis. J Immunol (1995) 154:5320–30. 7730635

[29] Xu M Mizoguchi I Morishima N Chiba Y Mizuguchi J Yoshimoto T. Regulation of antitumor immune responses by the IL-12 family cytokines, IL-12, IL-23, and IL-27. Clin Dev Immunol (2010) 2010:832454. 10.1155/2010/832454 20885915 PMC2946577

[30] Hunter CA. New IL-12-family members: IL-23 and IL-27, cytokines with divergent functions. Nat Rev Immunol (2005) 5:521–31. 10.1038/nri1648 15999093

[31] Kastelein RA Hunter CA Cua DJ. Discovery and biology of IL-23 and IL-27: related but functionally distinct regulators of inflammation. Annu Rev Immunol (2007) 25:221–42. 10.1146/annurev.immunol.22.012703.104758 17291186

[32] Ziblat A Domaica CI Spallanzani RG Iraolagoitia XL Rossi LE Avila DE IL-27 stimulates human NK-cell effector functions and primes NK cells for IL-18 responsiveness. Eur J Immunol (2015) 45:192–202. 10.1002/eji.201444699 25308526

[33] Zwirner NW Ziblat A. Regulation of NK cell activation and effector functions by the IL-12 family of cytokines: the case of IL-27. Front Immunol (2017) 8:25. 10.3389/fimmu.2017.00025 28154569 PMC5243847

[34] Lauwerys BR Renauld JC Houssiau FA. Synergistic proliferation and activation of natural killer cells by interleukin 12 and interleukin 18. Cytokine (1999) 11:822–30. 10.1006/cyto.1999.0501 10547269

[35] Oppmann B Lesley R Blom B Timans JC Xu Y Hunte B Novel p19 protein engages IL-12p40 to form a cytokine, IL-23, with biological activities similar as well as distinct from IL-12. Immunity (2000) 13(5):715–25. 10.1016/S1074-7613(00)00070-4 11114383

[36] Parham C Chirica M Timans J Vaisberg E Travis M Cheung J A receptor for the heterodimeric cytokine IL-23 is composed of IL-12Rbeta1 and a novel cytokine receptor subunit, IL-23R. J Immunol (2002) 168:5699–708. 10.4049/jimmunol.168.11.5699 12023369

[37] Gerosa F Baldani-Guerra B Lyakh LA Batoni G Esin S Winkler-Pickett RT Differential regulation of interleukin 12 and interleukin 23 production in human dendritic cells. J Exp Med (2008) 205:1447–61. 10.1084/jem.20071450 18490488 PMC2413040

[38] Sieve AN Meeks KD Lee S Berg RE A novel immunoregulatory function for IL-23: inhibition of IL-12-dependent IFN-gamma production. Eur J Immunol (2010) 40:2236–47. 10.1002/eji.200939759 20458705 PMC3039303

[39] Lankford CS Frucht DM. A unique role for IL-23 in promoting cellular immunity. J Leukoc Biol (2003) 73:49–56. 10.1189/jlb.0602326 12525561

[40] Teng MW Andrews DM McLaughlin N von Scheidt B Ngiow SF Moller A IL-23 suppresses innate immune response independently of IL-17A during carcinogenesis and metastasis. Proc Natl Acad Sci U S A (2010) 107:8328–33. 10.1073/pnas.1003251107 20404142 PMC2889517

[41] Hu J Yuan X Belladonna ML Ong JM Wachsmann-Hogiu S Farkas DL Induction of potent antitumor immunity by intratumoral injection of interleukin 23-transduced dendritic cells. Cancer Res (2006) 66:8887–96. 10.1158/0008-5472.CAN-05-3448 16951206

[42] Kaiga T Sato M Kaneda H Iwakura Y Takayama T Tahara H. Systemic administration of IL-23 induces potent antitumor immunity primarily mediated through Th1-type response in association with the endogenously expressed IL-12. J Immunol (2007) 178:7571–80. 10.4049/jimmunol.178.12.7571 17548592

[43] Rossi LE Avila DE Spallanzani RG Ziblat A Fuertes MB Lapyckyj L Histone deacetylase inhibitors impair NK cell viability and effector functions through inhibition of activation and receptor expression. J Leukoc Biol (2012) 91:321–31. 10.1189/jlb.0711339 22124136

[44] Chattopadhyay PK Yu J Roederer M. Live-cell assay to detect antigen-specific CD4+ T-cell responses by CD154 expression. Nat Protoc (2006) 1:1–6. 10.1038/nprot.2006.1 17406204

[45] Slinker BK The statistics of synergism. J Mol Cell Cardiol (1998) 30:723–31. 10.1006/jmcc.1998.0655 9602421

[46] Chaix J Tessmer MS Hoebe K Fuseri N Ryffel B Dalod M Priming of NK cells by IL-18. J Immunol (2008) 181:1627–31. 10.4049/jimmunol.181.3.1627 18641298 PMC5154249

[47] Lo CH Lee SC Wu PY Pan WY Su J Cheng CW Antitumor and antimetastatic activity of IL-23. J Immunol (2003) 171:600–7. 10.4049/jimmunol.171.2.600 12847224

[48] Langowski JL Zhang X Wu L Mattson JD Chen T Smith K IL-23 promotes tumour incidence and growth. Nature (2006) 442:461–5. 10.1038/nature04808 16688182

[49] Langrish CL Chen Y Blumenschein WM Mattson J Basham B Sedgwick JD IL-23 drives a pathogenic T cell population that induces autoimmune inflammation. J Exp Med (2005) 201:233–40. 10.1084/jem.20041257 15657292 PMC2212798

[50] van de Wetering D de Paus RA van Dissel JT van de Vosse E. IL-23 modulates CD56+/CD3- NK cell and CD56+/CD3+ NK-like T cell function differentially from IL-12. Int Immunol (2009) 21:145–53. 10.1093/intimm/dxn132 19088061

[51] Mailliard RB Alber SM Shen H Watkins SC Kirkwood JM Herberman RB IL-18-induced CD83+CCR7+ NK helper cells. J Exp Med (2005) 202:941–53. 10.1084/jem.20050128 16203865 PMC2213172

[52] Lucas M Schachterle W Oberle K Aichele P Diefenbach A. Dendritic cells prime natural killer cells by trans-presenting interleukin 15. Immunity (2007) 26:503–17. 10.1016/j.immuni.2007.03.006 17398124 PMC2084390

[53] Semino C Angelini G Poggi A Rubartelli A. NK/iDC interaction results in IL-18 secretion by DCs at the synaptic cleft followed by NK cell activation and release of the DC maturation factor HMGB1. Blood (2005) 106:60916. 10.1182/blood-2004-10-3906 15802534

[54] Borg C Jalil A Laderach D Maruyama K Wakasugi H Charrier S NK cell activation by dendritic cells (DCs) requires the formation of a synapse leading to IL-12 polarization in DCs. Blood (2004) 104:3267–75. 10.1182/blood-2004-01-0380 15242871

[55] Liu C Zhang Y Zhan J Zhao Y Wan Q Peng H Interleukin-23A is associated with tumor growth in *Helicobacter-pylori*-related human gastric cancer. Cancer Cell Int (2014) 14:104. 10.1186/s12935-014-0104-x 25349535 PMC4207902

[56] Nogueira E Hamour S Sawant D Henderson S Mansfield N Chavele KM Serum IL-17 and IL-23 levels and autoantigen-specific Th17 cells are elevated in patients with ANCA-associated vasculitis. Nephrol Dial Transplant (2010) 25:2209–17. 10.1093/ndt/gfp783 20100727

[57] Vaknin-Dembinsky A Balashov K Weiner HL IL-23 is increased in dendritic cells in multiple sclerosis and down-regulation of IL-23 by antisense oligos increases dendritic cell IL-10 production. J Immunol (2006) 176:7768–74. 10.4049/jimmunol.176.12.7768 16751425

[58] Roses RE Xu S Xu M Koldovsky U Koski G Czerniecki BJ. Differential production of IL-23 and IL-12 by myeloid-derived dendritic cells in response to TLR agonists. J Immunol (2008) 181:5120–7. 10.4049/jimmunol.181.7.5120 18802116

[59] Verreck FA de Boer T Langenberg DM Hoeve MA Kramer M Vaisberg E Human IL-23-producing type 1 macrophages promote but IL-10-producing type 2 macrophages subvert immunity to (myco)bacteria. Proc Natl Acad Sci U S A (2004) 101:4560–5. 10.1073/pnas.0400983101 15070757 PMC384786

[60] Kondadasula SV Roda JM Parihar R Yu J Lehman A Caligiuri MA Colocalization of the IL-12 receptor and FcgammaRIIIa to natural killer cell lipid rafts leads to activation of ERK and enhanced production of interferon-gamma. Blood (2008) 111:4173–83. 10.1182/blood-2007-01-068908 18174382 PMC2288725

[61] Nandagopal N Ali AK Komal AK Lee SH. The critical role of IL-15-PI3K-mTOR pathway in natural killer cell effector functions. Front Immunol (2014) 5:187. 10.3389/fimmu.2014.00187 24795729 PMC4005952

[62] Hao JS Shan BE. Immune enhancement and anti-tumour activity of IL-23. Cancer Immunol Immunother (2006) 55:1426–31. 10.1007/s00262-006-0171-5 16676182 PMC11031071

[63] Pandya AD Al-Jaderi Z Hoglund RA Holmoy T Harbo HF Norgauer J Identification of human NK17/NK1 cells. PLoS One (2011) 6:e26780. 10.1371/journal.pone.0026780 22039549 PMC3198820

[64] Perona-Wright G Mohrs K Szaba FM Kummer LW Madan R Karp CL Systemic but not local infections elicit immunosuppressive IL-10 production by natural killer cells. Cell Host Microbe (2009) 6:503–12. 10.1016/j.chom.2009.11.003 20006839 PMC2796259

[65] Carbone T Nasorri F Pennino D Donnarumma M Garcovich S Eyerich K CD56 highCD16- NK cell involvement in cutaneous lichen planus. Eur J Dermatol (2010) 20:724–30. 10.1684/ejd.2010.1096 20959273

[66] Kubota A Lian RH Lohwasser S Salcedo M Takei F. IFN-gamma production and cytotoxicity of IL-2-activated murine NK cells are differentially regulated by MHC class I molecules. J Immunol (1999) 163:6488–93. 10586040

[67] Agaugué S Marcenaro E Ferranti B Moretta L Moretta A. Human natural killer cells exposed to IL-2, IL-12, IL-18, or IL-4 differently modulate priming of naive T cells by monocyte-derived dendritic cells. Blood (2008) 112:1776–83. 10.1182/blood-2008-02-135871 18579793

[68] Carbone E Terrazzano G Ruggiero G Zanzi D Ottaiano A Manzo C Recognition of autologous dendritic cells by human NK cells. Eur J Immunol (1999) 29:4022–9. 10.1002/(SICI)1521-4141(199912)29:12<4022::AID-IMMU4022>3.0.CO;2-O 10602012

[69] Carbone E Ruggiero G Terrazzano G Palomba C Manzo C Fontana S A new mechanism of NK cell cytotoxicity activation: the CD40-CD40 ligand interaction. J Exp Med (1997) 185:2053–60. 10.1084/jem.185.12.2053 9182676 PMC2196353

